# Lactate secreted by PKM2 upregulation promotes Galectin-9-mediated immunosuppression via inhibiting NF-κB pathway in HNSCC

**DOI:** 10.1038/s41419-021-03990-4

**Published:** 2021-07-21

**Authors:** Hanyue Chang, Qiaoshi Xu, Jiayi Li, Mingyu Li, Zhiyuan Zhang, Hailong Ma, Xi Yang

**Affiliations:** 1 Department of Oral Maxillofacial-Head and Neck Oncology, Shanghai Ninth People’s Hospital, College of Stomatology, Shanghai Jiao Tong University School of Medicine, No 639, Zhizaoju Rd, 200011 Shanghai, China; 2 National Clinical Research Center for Oral Diseases, 200011 Shanghai, China; 3 Shanghai Key Laboratory of Stomatology & Shanghai Research Institute of Stomatology, 200011 Shanghai, China; 4 Key Laboratory of Oral Medicine, Guangzhou Institute of Oral Disease, Stomatology Hospital of Guangzhou Medical University, 510140 Guangzhou, China

**Keywords:** Cancer metabolism, Tumour immunology

## Abstract

Pyruvate kinase M2 as a key rate-limiting enzyme in glycolysis, it plays a critical role in metabolic reprogramming and carcinogenesis. However, whether PKM2 can promote immunosuppressive microenvironment formation remains unknown in head and neck squamous cell carcinoma (HNSCC). PKM2 expression was detected using immunohistochemical staining. The biological functions of PKM2 were investigated in vitro and in vivo. Lactate production and the expression of Galectin-9, a critical immunosuppression molecule, were detected after PKM2 knockdown and overexpression in HNSCC cells. The mechanism of lactate regulating Galectin-9 expression through NF-κB signaling was explored in vitro. Overexpression of PKM2 correlates with poor prognosis in HNSCC patients. Silencing PKM2 markedly inhibits proliferation and metastasis capacity in vivo and in vitro, and vice versa. The glycolysis and glycolytic capacity are significantly decreased after PKM2 silencing. Lactate secretion induced by PKM2 significantly promotes migration and invasion capacity. Furthermore, a positive correlation between PKM2 and Galectin-9 expression is observed in HNSCC tissues. The induction of Galectin-9 expression by PKM2 can be affected by a lactate transporter inhibitor. Mechanically, lactate impeded the suppressive transcriptional complex formation of NF-κB and histone deacetylase 3 (HDAC3), which released the transcription of Galectin-9 mediated by NF-κB signaling. Our findings demonstrate that lactate produced by PKM2 upregulation promotes tumor progression and Galectin-9-mediated immunosuppression via NF-κB signaling inhibition in HNSCC, which bridges metabolism and immunosuppression. The novel PKM2-lactate-Galectin-9 axis might be a potential therapeutic target in HNSCC.

## Introduction

Head and neck cancer is the eighth most common cause of cancer death worldwide [1] and head and neck squamous cell carcinoma (HNSCC) accounts for most of those [2]. With the approvals of anti-PD-1 immune checkpoint inhibitors, immunotherapeutic strategies have largely used for the treatment of HNSCC patients. However, only 15% of patients can benefit from immunotherapy, it is urgent to deeply investigate immunosuppression mechanism [3]. Immunosuppression facilitates tumor immune escape by inhibiting antitumor immune responses, which plays a critical role in malignant progression and tumor metastasis [4]. Immunoevasion is an emerging hallmark of cancer [5]. Galectin‑9, a member of the β‑galactoside lectin family, is involved many processes of T cell‑mediated diseases and immunomodulation of macrophages [6]. It is a novel and critical checkpoint molecule on tumor surfaces that binds to T cell immunoglobulin and mucin domain-containing protein 3 (TIM-3) on immune cells to inhibit the killing effect of immune cells [7]. Tumor microenvironment acidification drives immune escape by destroying the killing function of T and natural killer (NK) cells [8]. It means cancer cells could escape from immune surveillance by increasing lactate output [9]. How metabolite such as lactate regulate immunosuppression remains unknown.

Lactate accumulation is a characteristic of tumor microenvironment. As we all know, the energy metabolism capacity of tumor cells is characterized by the Warburg effect, such that even in the presence of oxygen, cancer cells increase glucose uptake and conversion to lactic acid, which is termed “aerobic glycolysis” [10]. Energy reprogramming, another hallmarks of cancer, shows that tumors have to adjust their energy metabolism in order to fuel cell growth [5]. Pyruvate kinase is a kinase that physiologically irreversibly catalyzes phosphoenolpyruvate to produce pyruvate as the last step of glycolysis [11]. The embryonic M2 isoform of pyruvate kinase (PKM2) is exclusively expressed in tumor tissues [12]. Nowadays the crosstalk between immunosuppression and metabolites is a prominent research area. Function and regulation in immunosuppression formation of PKM2 requires further investigation.

In this study, we demonstrate that overexpression of PKM2 can drive tumor immunosuppression formation and progression through lactate secretion in HNSCC. Moreover, lactate impeded the suppressive transcriptional complex formation of NF-κB and histone deacetylase 3 (HDAC3), which released the transcription of Galectin-9 mediated by NF-κB signaling. Therefore, our results indicate that lactate secreted by PKM2 significantly promotes Galectin-9-mediated immunosuppression through NF-κB pathway inhibition, which bridges metabolites with immunosuppression and indicates a promising therapeutic target in HNSCC.

## Materials and methods

### Patient and tissue samples

We collected 70 HNSCC tissue samples and 18 healthy subjects from January 2007 to December 2008. Informed consent was obtained for all participants. This study was approved by the Ethics Committee of the Ninth People’s Hospital, Shanghai Jiao Tong University School of Medicine and that it conformed to the provisions of the Declaration of Helsinki.

### Cell culture

The HNSCC cell lines Cal27, HN4, HN6, HN30, SCC4, and SCC25, along with primary normal tongue epithelia cells, were used in this study. The cell culture condition and source were described in our previous study. Lactate was purchased from Sigma-Aldrich (St. Louis, MO, USA). Compound 3K (a PKM2 inhibitor), TEPP46 (a PKM2 activator), RFPG966 (the specific inhibitor of HDAC3), and AZD3965 (a MCT1 inhibitor) were from Selleck (Houston, TX, USA).

### Cell transfection

Small interfering RNA (siRNA) for PKM2 (RiboBio, Guangzhou, China) and relative expressing plasmids (BioLink, Shanghai, China) were transiently transfected into HNSCC cell lines using Lipofectamine™ 3000 (Invitrogen, Carlsbad, CA, USA) according to the manufacturer’s instructions. The sequences of PKM2 siRNA were: #1, 5′-CCAACACCATGCGTGTTGT-3′, #2, 5′-GGATGTTGATATGGTGTTT-3′, and #3, 5′-GTGGTGATCTAGGCATTGA-3′. The #2 sequence was chosen for the lentivirus package since it has perfect silencing efficiency. The siRNA sequences of Galectin-9 were 5′-GAGGGUACGUGGUGUGCAA-3′.

### Immunohistochemistry

Immunohistochemical (IHC) staining was strictly performed according to our previous study. Rabbit anti-human PKM2 (CST, Danvers, MA, USA), Galectin-9 (CST, Danvers, MA, USA), Lactate Dehydrogenase A (LDHA) (CST, Danvers, MA, USA), E-cadhein (CST, Danvers, MA, USA), and ki-67 (Proteintech, Rocky Hill, NJ, USA) antibodies were overnight incubated and visualized (Dako Cytomation, Denmark). The immunoreaction score (IRS) was calculated by multiplying the percentage of positive cells and the staining intensity as described in the literature [13].

### Cellular proliferation capacity assay

Cell Counting Kit-8 (CCK8, Dojindo, Kumamoto, Japan), colony formation, and EdU assays were used to analyze cell growth capacity, as described in our previous study [14]. The EdU assay was performed with the Cell-Light EdU Apollo in vitro Imaging Kit (Ribobio, Guangzhou, China) according to the manufacturer’s instructions.

### Cell migration and invasion assay

Migration ability was examined by both a wound healing assay and a transwell assay (uncoated insert). 1–6 × 10^4^ cells were seeded in BioCoat™ inserts (BD Biosciences, Franklin Lake, NJ, USA) for invasion. The migrating and invading cells were stained using 5% crystal violet. All the experiments were performed in triplicate.

### Real-time PCR assay

TRIzol Reagent (Takara, Japan) was used to extract total RNA. PKM2 mRNA expression was quantified using the SYBR Premix Ex Taq Reagent Kit (Takara, Japan) on an ABI StepOne real-time PCR system (Applied Biosystems, USA) as described in our previous study [15]. The PKM2 primer sequences were 5′-TGACGAGAACATCCTGTGGC-3′ (forward), 5′-TTTTCCACCTCCGTCACCAG-3′ (reverse), and GAPDH sequences were 5′-CCTCTGACTTCAACAGCGAC-3′ (forward), 5′-TCCTCTTGTGCTCTTGCTGG-3′ (reverse).

### Immunoblotting and immunoprecipitation analysis

The cell protein was extracted in SDS lysis buffer (Beyotime, Shanghai, China). The protein samples were electrophoresed on 4–20% polyacrylamide gels (Genshare Biological, China) and transferred to polyvinylidene fluoride (PVDF) membranes (Merck Millipore, USA). The antibodies for PKM2, p-AKT (Ser473), AKT, p-ERK1/2 (Thr202/Tyr204), ERK1/2, Galectin-9, E-cadherin, MMP2, MMP9, p-IKKα/β (Ser176/180), IKKα/β, p-IκBα (Ser32), IκBα, p-NF-κB p65 (Ser536), NF-κB p65, p-AMPK (Thr172), AMPK, p-LATS1 (Thr1079), LATS1, and p-YAP(Ser127) were purchased from Cell Signaling Technology (Danvers, MA, USA). β-actin, α-tubulin and GAPDH antibodies (Proteintech, Rocky Hill, NJ, USA) were used as internal controls. For immunoprecipitation analysis, Cells were lysed with RIPA lysis (Beyotime, Shanghai, China), incubated with specific antibodies and normal IgG and followed by incubation with Protein A/G Magnetic Beads (Bimake, Houston, TX, USA). Immunoreactive bands were visualized with ECL Ultra (New Cell and Molecular Biotech, Suzhou, China).

### Chromatin immunoprecipitation

The chromatin immunoprecipitation (ChIP) assay was performed according to the manufacturer’s instructions of SimpleChIP Enzymatic Chromatin IP kit (CST, Danvers, MA, USA) The chromatin of treated cells was fixed, lysed and sonicated. The binding DNA fragments were analyzed by qPCR. NF-κB p65 antibody and IgG (CST, Danvers, MA, USA) was used to enrich the promoter regions of Galectin-9.

### Dual-luciferase reporter assay

293T cells were cotransfected with promoter-luciferase plasmids and pRL-TK Renilla luciferase construct as an internal control (30:1). After 36 h, the cells were harvest in lysis buffer and luciferase activity was detected using a dual-luciferase reporter assay system (Beyotime, Shanghai, China).

### Immunofluorescence, Actin-Tracker Green staining, and TUNEL assay

After fixation, cells were incubated with primary antibodies against NF-κB p65 and then secondary antibodies. Cells were costained with DAPI (Beyotime, Shanghai, China) and then visualized using a fluorescence microscope (Zeiss, Jena, Germany) and a laser scanning confocal microscope (Zeiss, Jena, Germany). Cell morphology was detected by Actin-Tracker Green staining kit (Beyotime, Shanghai, China) under an AxioVert A1 fluorescence microscope. Cell activity was detected according to instructions of Calcein AM Cell Viability Assay Kit (Beyotime, Shanghai, China). Dead cells were dyed red and observed under fluorescence microscope (Zeiss, Jena, Germany). The Terminal deoxynucleotidyl transferase dUTP nick end labeling (TUNEL) assay was performed following the manufacturer’s instructions (Beyotime, Shanghai, China).

### Enzyme-linked Immunosorbent assay

Galectin-9 levels in medium supernatant were quantified using an enzyme-linked immunosorbent assay (ELISA) kit (R&D, Minneapolis, MN). IL-1β and IL-6 levels in medium supernatant were quantified using ELISA kit (Multisciences, Hangzhou, China). The supernatant lactate was measured by a lactate assay kit (Sigma-Aldrich, St. Louis, MO, USA).

### Flow cytometry

A FITC Annexin V Apoptosis Detection Kit (BD Biosciences, Franklin Lakes, NJ, USA) was used for detecting cell apoptosis. PI/RNase staining buffer (BD Biosciences, Franklin Lakes, NJ, USA) was used for detecting cell cycle distribution. Murine tumors were harvested and processed into single cell suspensions via mechanical dissociation, filtration, and lysis of erythrocytes. After tumor was processed into single cell suspensions, surface staining was performed using CD11b-FITC, Gr-1-APC, and Ly6G-PE antibodies (Proteintech, Rocky Hill, NJ, USA) and detected on a BD Beckman cytometer (BD Biosciences, Franklin Lakes, NJ, USA).

### Natural killer cell lysis assay

The transfected cells were cocultured with NK cells at different effector-to-target (E:T) cell ratios as indicated for 4 h. The cytotoxicity of NK cells was assessed using a Lactate Dehydrogenase (LDH) Cytotoxicity Assay Kit (Beyotime, Shanghai, China) following the manufacturer’s instructions.

### Metabolic flux assay

The extracellular acidification rate was analyzed on an XF96 Extracellular Flux Analyzer (Agilent Technologies, Sana Clara, USA). Cells were seeded in XFe 96-well microplates (10000 cells/well) for 24 h, washed and incubated in XF RPMI medium at 37 °C for 1 h. Extracellular acidification Rate (ECAR) were measured in response to 25 mM glucose, 1 μM oligomycin, and 50 mM 2-deoxy-d-glucose in real-time conditions.

### Animal studies

BALB/c nude mice were purchased from the Shanghai Laboratory Animal Center (Shanghai, China). For the xenograft model, 1 × 10^6^ Cal27 cells were injected into the flank of the mouse. The mice were injected with RGFP966 (10 mg/kg/day, i.p) or physiological saline. For the lung metastasis model, 1 × 10^6^ HN30 cells were intravenously injected into the tail vein. The lungs were fixed in Bouin’s fixative diluted 1:5 with neutral buffered formalin. The animal studies were approved by the Animal Care and Use Committee of Ninth People’s Hospital, Shanghai Jiao Tong University School of Medicine.

### Statistical analysis

Statistical analyses were performed with SPSS 13.0 for Windows (SPSS Inc., Chicago, IL) and GraphPad Prism version 8 (GraphPad Software, San Diego, CA, USA). Student’s *t* test were used to compare two group. One-way ANOVA followed by Tukey’s honestly significant difference test was used to assess more than three groups. *P* values < 0.05 were considered significant (*P < 0.05, and **P < 0.01). The data are presented as the means ± SD.

## Results

### Overexpression of PKM2 correlates with poor prognosis in HNSCC patients

**Fig. 1:**
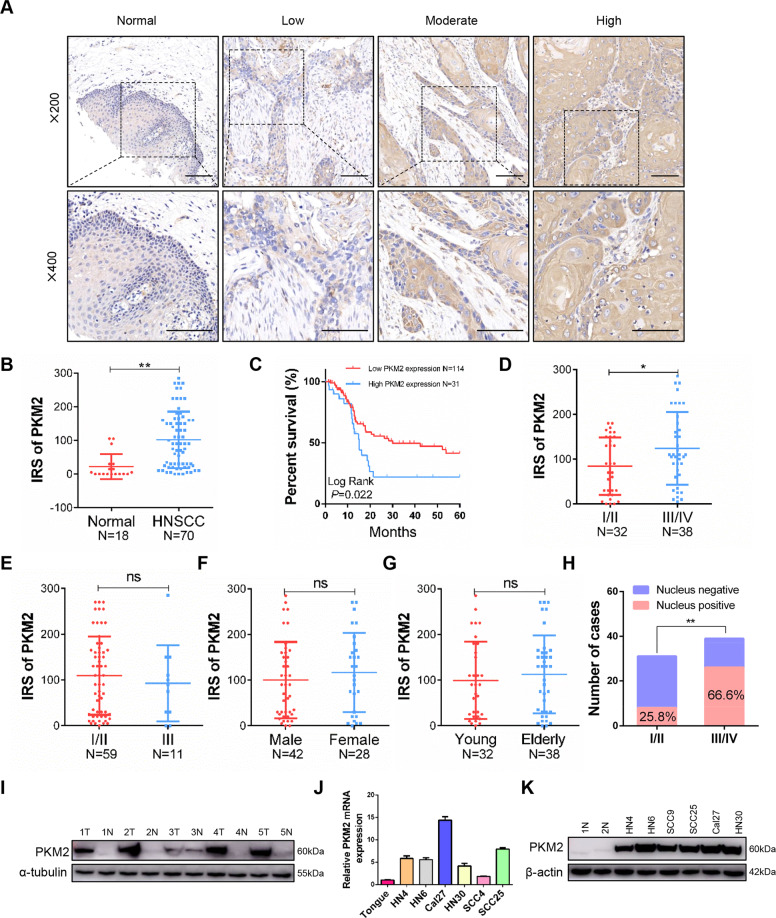
Overexpression of PKM2 correlates with poor prognosis. **A** Representative images of high, moderate, and low expression of PKM2 in HNSCC were shown. Bar: 50 μm. **B** IHC score of PKM2 in HNSCC patients (*n* = 70) and normal controls (*n* = 18) was analyzed. **c** Overall survival analysis was performed based on PKM2 expression in 144 HNSCC patients. **D**–**G** The correlation between PKM2 expression and TNM stage, pathological grade, gender, and age was analyzed in HNSCC patients. **H** The correlation between nuclear PKM2 expression and TNM stage was analyzed in HNSCC. **I** PKM2 protein levels were determined in 5 paired HNSCC samples using western blotting (N adjacent normal tissue, T tumor tissue). **J** PKM2 mRNA expression in HNSCC cell lines and primary normal oral keratinocytes was detected using qRT-PCR. **K** The relative expression of PKM2 in HNSCC cell lines and normal oral epithelial cells as determined by western blotting. *P < 0.05, **P < 0.01.

Firstly, we detected the PKM2 expression in HNSCC tissues and normal oral mucosa using immunohistochemical staining. We observed that PKM2 was mainly expressed in cytoplasm (Fig. 1A). The IRS of PKM2 was significantly higher in HNSCC samples than normal samples (Fig. 1B, *P* < 0.01). Furthermore, we found that HNSCC patients with high PKM2 expression had worse outcomes in The Cancer Genome Atlas (TCGA) dataset (Fig. 1C, *P* = 0.022). The expression level of PKM2 correlated with advanced TNM stage (Fig. 1D, *P* = 0.0426). However, there was no correlation between PKM2 expression and other factors, including pathological grade (Fig. 1E), gender (Fig. 1F) and age (Fig. 1G). It is reported that nuclear translocation of PKM2 is implicated in several cancer models. Thus, we detected the nuclear PKM2 expression in HNSCC tissues and the expression of nuclear PKM2 correlated with advanced TNM stage (Fig. S1). Patients with TNM I/II stage had a nucleus positive percentage of 25.8% (8/31), while 66.6% (26/39) in TNM III/IV stage (Fig. 1H). Moreover, PKM2 was higher expressed in fresh tumor tissues compared with adjacent normal tissues (Figs. 1I and S2). We observed that the mRNA and protein level of PKM2 was upregulated in HNSCC cell lines and normal primary oral keratinocytes (Figs. 1J, K, and S3). These results demonstrated that the expression of PKM2 in HNSCC was increased and correlated with poor prognosis.

### PKM2 enhances cell proliferation capacity in HNSCC cells

**Fig. 2:**
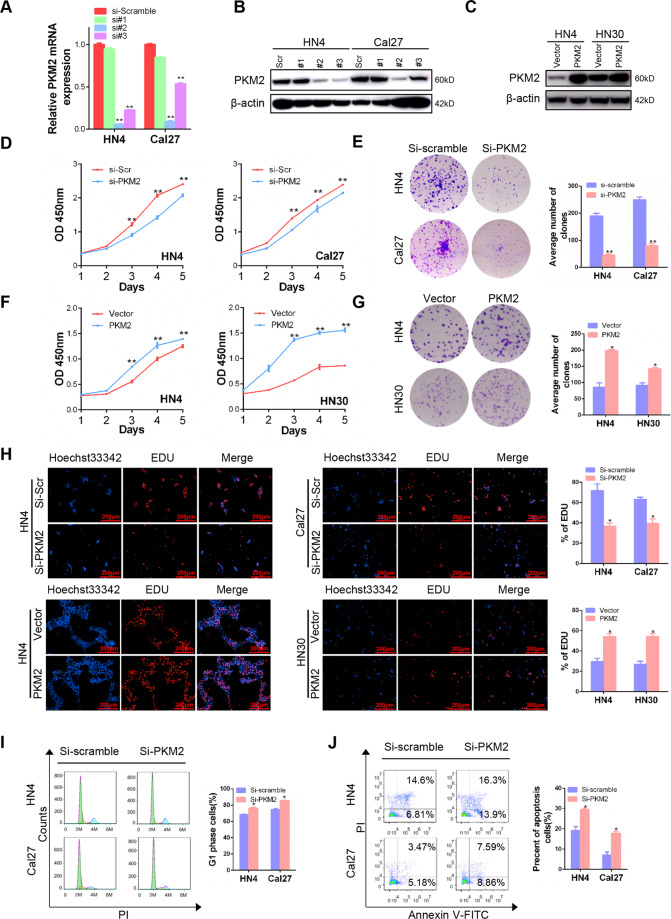
PKM2 enhances cell proliferation capacity in HNSCC cells. **A**, **B** Transfection efficiency was detected using PCR and western blot after PKM2-specific siRNA transfection for 48 h in HN4 and Cal27 cells. **C** PKM2 expression was detected after plasmid transfection for 48 h in HN4 and HN30 cells. **D**–**H** The CCK8, colony formation, and EdU assay were performed to detect proliferation ability after PKM2-specific siRNA transfection for 24 h in HN4 and Cal27 cells or PKM2 plasmid transfection for 24 h in HN4 and HN30 cells. **I** The cell cycle was analyzed using PI staining after siRNA transfection for 48 h. **J** The proportion of apoptotic cells (lower and upper right fraction) after transfection with siRNA for 48 h was analyzed using PI and Annexin V staining. *P < 0.05, **P < 0.01.

To further explore the biological role of PKM2 in OSCC cells, silencing and ectopic expression models were established in HNSCC cell lines (Fig. 2A–C). Knockdown of PKM2 significantly inhibited cell proliferation, colony formation, and DNA replication capacity compared to the scramble control (Fig. 2D, E, H). To the contrary, overexpression PKM2 promoted cell proliferation, colony formation, and DNA replication (Fig. 2F–H). Moreover, knockdown of PKM2 promoted G1 arrest and induced apoptosis in HN4 and Cal27 (Fig. 2I, J). Furthermore, we detected the proliferation-related signaling to observe that knockdown of PKM2 expression increased the level of p-ERK1/2, but not AKT, in both HN4 and Cal27 cells (Fig. S4). Conversely, overexpression of PKM2 inhibited p-ERK1/2 expression in HN4 and HN30 cells (Fig. S4). These results indicated that overexpression of PKM2 notably promoted proliferation capacity in HNSCC cells.

### PKM2 promotes migration and invasion in HNSCC cells

**Fig. 3:**
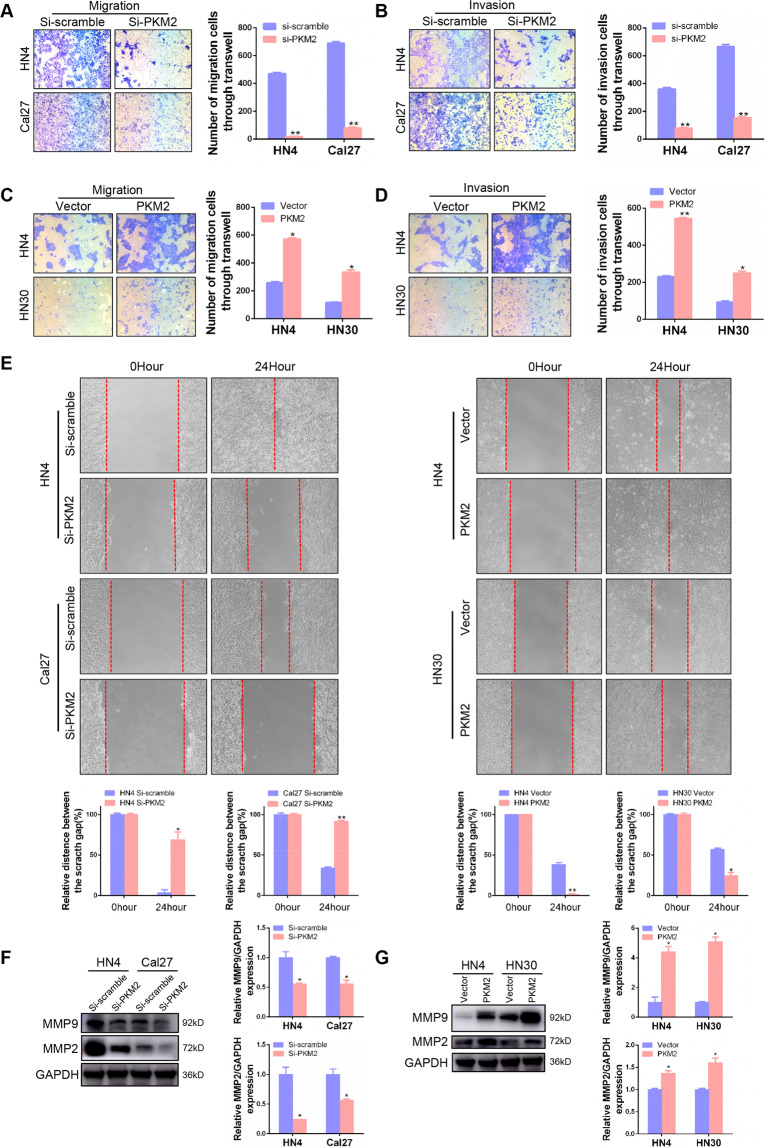
PKM2 promotes migration and invasion in HNSCC cells. **A**, **B** Migration and invasion ability was studied using a transwell assay after siRNA transfection for 24 h in HN4 and Cal27 cells. **C**, **D** Migration and invasion ability was performed using the transwell assay after plasmid transfection for 24 h in HN4 and HN30 cells. **E** A wound healing assay was conducted to detect the migration ability of HNSCC cells after PKM2-specific siRNA or plasmid transfection for 24 h. **F**, **G** MMP9 and MMP2 were detected after the indicated transfection for 48 h. *P < 0.05, **P < 0.01.

In addition, the proliferation, migration, and invasion capacity were also explored. The number of invasion and migration cells was significantly decreased after PKM2 knockdown in HN4, Cal27, and HN30 cells (Fig. 3A, B and S5). However, ectopic expression of PKM2 enhanced migration and invasion in HN4 and HN30 cells (Fig. 3C, D). The wound healing assay also showed that cells after PKM2 silencing migrated more slowly than the scramble control, and the opposite was true for cells overexpressing PKM2 (Fig. 3E). Furthermore, MMP9 and MMP2, two metastasis-related proteins, were inhibited after PKM2 knockdown (Fig. 3F), while they increased after upregulation of PKM2 (Fig. 3G). These results demonstrated that PKM2 could accelerate the aggressive progression in HNSCC cells, though the mechanism needs further investigation.

### Lactate released by PKM2 overexpression promotes migration and invasion in HNSCC

**Fig. 4:**
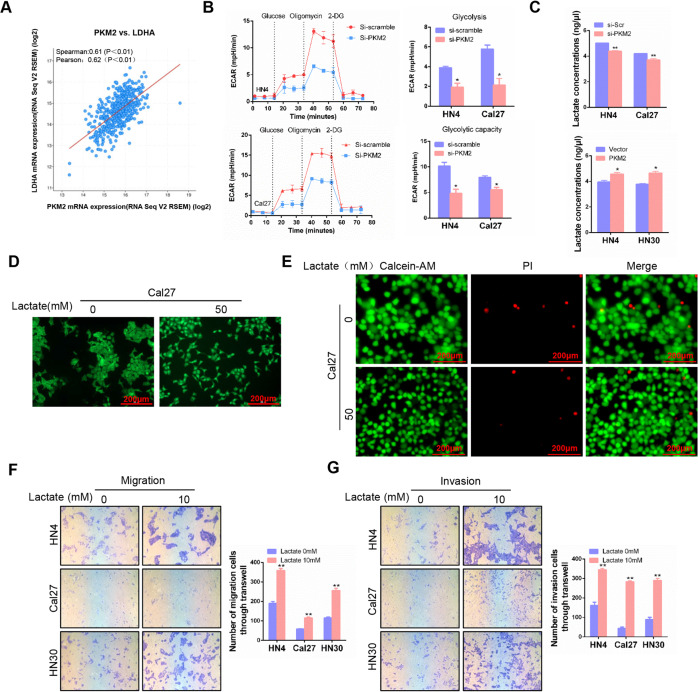
Lactate released by PKM2 overexpression promotes migration and invasion in HNSCC. **A** The correlation between PKM2 and LDHA in HNSCC datasets from the TCGA database was analyzed. **B** The real-time ECAR was analyzed by Seahorse method after PKM2-siRNA transfection for 48 h. **c** Lactate concentration was detected in medium supernatant after transfection with PKM2 siRNA or PKM2 expression plasmids for 48 h. **D** The morphology of Cal27 was detected by Actin-Tracker Green after 50 mM lactate stimulation for 24 h. **E** Live/dead cell staining of was analyzed in Cal27 after treated with 50 mM lactate for 24 h. **F**, **G** Migration and invasion assays were performed using the transwell assay after 10 mM lactate treatment for 24 h. bar: 200 μm *P < 0.05, **P < 0.01.

A positive correlation between PKM2 and LDHA was observed in the HNSCC dataset from the TCGA database (Fig. 4A). PKM2 was a critical enzyme in aerobic glycolysis, and we implemented a real-time ECAR analysis by Seahorse platform after PKM2 transfection. The glycolysis and glycolytic capacity were significantly decreased after PKM2 silencing in HN4 and Cal27 cell lines (Fig. 4B). The pH was decreased after PKM2 overexpression (Fig. S6). Then, we found that the lactate concentration in medium supernatant was increased after PKM2 upregulation, while it decreased after PKM2 knockdown (Fig. 4C). Interestingly, we observed that the cell morphology was dramatically altered from a typical cobblestone appearance into a star-like or shuttle shape (Figs. 4D and S7), which indicated that lactate might promote the epithelial-to-mesenchymal transition in HNSCC cells. To exclude the cell viability of lactate, live cell analysis was analyzed. Dead cells were stained red, while living cells were stained green. After treated with lactate, the number of red cells did not increase. It confirmed that lactate could directly alter the shape of HNSCC cells, which not affect the cell viability (Fig. 4E and S8). Mechanistically, 50 mM lactate could significantly inhibit the expression of E-cadherin, a specific epithelial marker (Fig. S9). Then, transwell assay revealed that lactate promoted cell migration and invasion capacity in HN4, Cal27, and HN30 cells (Fig. 4F, G). These results indicated that overexpression of PKM2 enhanced the secretion of lactate, which promoted migration and invasion in HNSCC cells.

### PKM2 promotes Galectin-9-mediated immunosuppression through lactate secretion

**Fig. 5:**
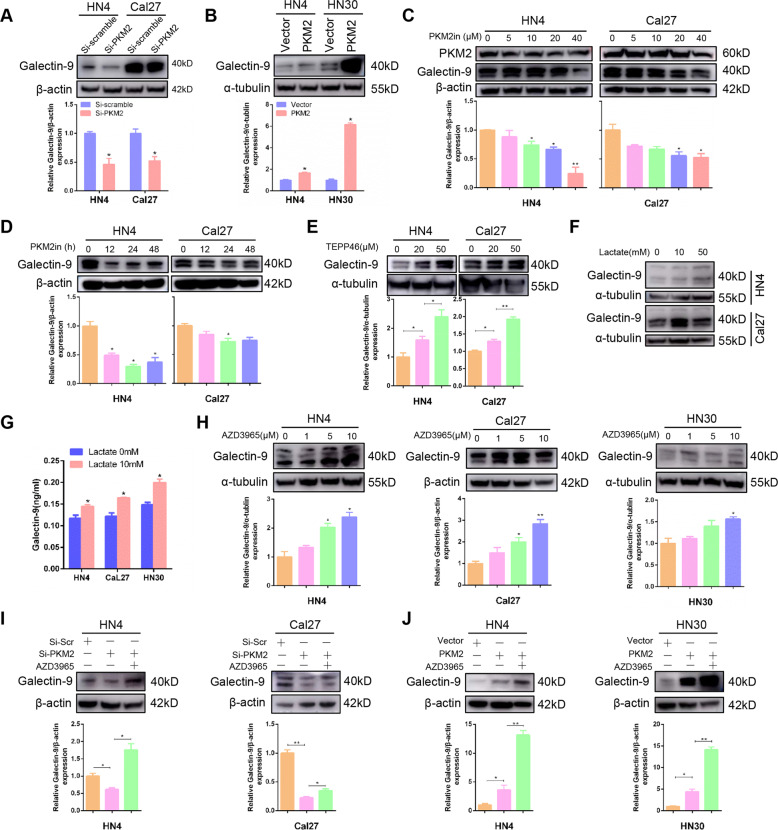
PKM2 promotes Galectin-9-mediated immunosuppression through lactate secretion. **A** Galectin-9 expression was detected after PKM2-specific siRNA transfection in HN4 and Cal27 cells for 48 h. **B** Galectin-9 expression was detected after transfection with PKM2 expressing plasmid transfection for 48 h. **C** Galectin-9 expression was detected after treatment with Compound 3K, a PKM2 inhibitor, at the indicated doses for 24 h. **D** Galectin-9 expression was detected after 40 μM Compound 3K treatment at the indicated times. **E** Galectin-9 expression was detected after treatment with TEPP46, a PKM2 activator, at the indicated doses for 24 h. **F** After lactate stimulation for 24 h, Galectin-9 expression was detected using western blot. **G** Galectin-9 concentration in medium supernatant after 10 mM lactate treatment for 24 h was detected using ELISA. **H** Galectin-9 was assessed after treatment with the indicated dose of AZD3965, a MCT1 inhibitor, for 24 h. **I**, **J** Galectin-9 was detected using western blot after PKM2 transfection for 24 h and then 10 μM AZD3965 treatment for 24 h. *P < 0.05, **P < 0.01.

As Galectin-9 was known as an immunosuppression molecule. We found that PKM2 knockdown inhibited Galectin-9 expression in HN4 and Cal27 cells (Fig. 5A), while ectopic expression of PKM2 promoted it (Fig. 5B). Compound 3K, a PKM2 inhibitor, inhibited Galectin-9 expression in a dose- and time-dependent manner in HN4 and Cal27 cells (Fig. 5C, D). TEPP46, activator of PKM2, increased Galectin-9 expression in a dose-dependent manner in HN4 and Cal27 cells (Fig. 5E). Furthermore, lactate stimulation can not only promote Galectin-9 expression in a dose-dependent manner (Figs. 5F and S10) but also its secretion (Fig. 5G). Consistent with the results, overexpression of PKM2 also increased the concentration of Galectin-9 in cell supernatants (Fig. S11). To further explore the role of lactate in PKM2-induced Galectin-9 expression, AZD3965, an inhibitor of monocarboxylate transporter 1 (MCT1), was used in our study. AZD3965 increased intracellular lactate and significantly reduced lactate intake [16, 17]. AZD3965 can significantly induce Galectin-9 expression in a dose-dependent manner in HN4, Cal27, and HN30 (Fig. 5H). AZD3965 can reverse the downregulation of Galectin-9 mediated by PKM2 silencing in HN4 and Cal27 cells (Fig. 5I), while synergistically increasing Galectin-9 expression after ectopic expression of PKM2 (Fig. 5J). These results demonstrated that PKM2 promoted Galectin-9 expression through lactate secretion in HNSCC. To further verify the immune function of Galectin-9, a knockdown model was established (Fig. S12). We found that knockdown of the Galectin-9 level notably enhanced the killing effect of NK cells (Fig. S13). Moreover, we also found that overexpression of PKM2 significantly increased the secretion of proinflammatory cytokines, IL-1β and IL-6, which might be involved in tumorigenesis and immunosuppression mediated by Galectin-9 (Fig. S14). These findings suggested that PKM2 promoted Galectin-9-mediated immunosuppression through lactate secretion in HNSCC.

### Lactate-induced Galectin-9 expression through inhibition of NF-κB signaling

**Fig. 6:**
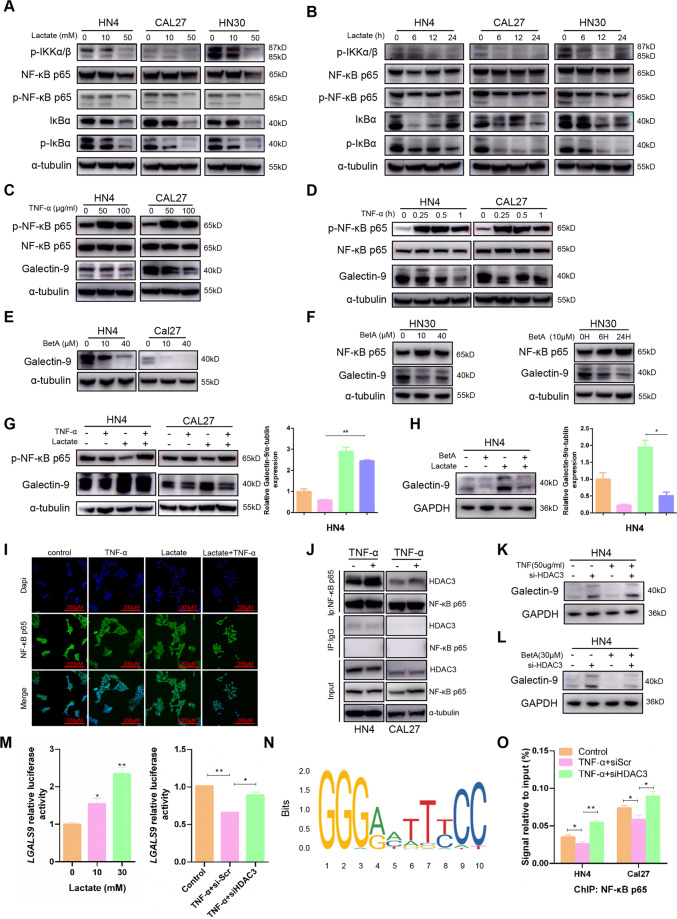
Lactate-induced Galectin-9 expression through inhibition of NF-κB signaling. **A**, **B** p-IKKα/β, NF-κB p65, and IκBα expression were detected after lactate stimulation in HNSCC cells for 24 h or 10 mM. The protein levels of Galectin-9 was detected after treatment of HN4 and Cal27 cells with TNF-α for 1 h (**C**) or 50 μg/ml (**D**). **E** After treatment with BetA, Galectin-9 was assessed using western blot. **F** The protein levels of NF-κB p65 and Galectin-9 were detected after treatment of HN30 cells with BetA. **G** After treatment with 50 μg/ml TNF-α and 10 mM lactate in HN4 and Cal27 cells, NF-κB p65 and Galectin-9 was assessed using western blot. **H** The expression of NF-κB p65 and Galectin-9 was determined in HN4 cells after incubation with 10 μM BetA and 10 mM lactate. **I** An immunofluorescence assay was used to visualize the localization of NF-κB p65 after treatment with 50 μg/ml TNF-α and 10 mM lactate. **J** Immunoprecipitation assay was performed using anti-NF-κB p65 antibody or IgG in HN4 and Cal27 cells after treatment with 50 μg/ml TNF-α. After HDAC3-specific siRNA transfection for 24 h, HN4 cells were treated with 50 μg/ml TNF-α for 15 min (**K**) or 10 μM BetA (**L**) for 24 h and then Galectin-9 was analyzed. **M** Transcriptional activity was detected using dual-luciferase reporter assays with *LGALS9* promoter-luciferase plasmids after treatment with 10 mM lactate or 10 μM BetA and HDAC3-specific siRNA in 293T cells. **N** The binding sites of NF-κB p65 in the Galectin-9 promoter was predicted according to JASPAR database. **o** ChIP assays were performed after treatment with 50 μg/ml TNF-α and HDAC3-specific siRNA. *P < 0.05, **P < 0.01.

We sought to determine the transcription factors involved in lactate-mediated Galectin-9 expression. Accumulating studies have reported that NF-κB play an essential role in inflammation and innate immunity [18], which suggested that NF-κB might be involved in lactate-induced Galectin-9 expression. To verify this hypothesis, the phosphorylation of IκBα, IKKα/β, and NF-κB p65 significantly attenuated after lactate treatment in a dose- and time-dependent manner, which indicated notably suppression of NF-κB signaling (Fig. 6A, B). Furthermore, we also detected the upstream kinases of NF-κB, such as AMPK, LATS, YAP, to find that lactate increased the level of p-AMPK, p-LATS, and p-YAP (Fig. S15). The phosphorylation of AMPK and YAP could inhibit the NF-κB signaling [19, 20], which might explain why lactate inhibited the activation of NF-kB pathway. Moreover, Galectin-9 is suppressed in a dose- and time-dependent manner after incubation with TNF-α, a well-known NF-κB activator (Fig. 6C, D). Betulinic acid, a specific activator of NF-κB, obviously inhibited the expression of Galectin-9 in HN4 and Cal27 cells (Fig. 6E). Meanwhile, we used different concentration of BetA to stimulate HN30 cells and observed a dose-dependent decrease of Galectin-9. The effects were also detected in a time-dependent manner in HN30 cells (Fig. 6F). Lactate and TNF-α was used in HN4 and Cal27 cells to illustrate the role of NF-κB in lactate-mediated Galectin-9. These data indicated that both TNF-α and BetA could inhibit Galectin-9 expression induced by lactate in HN4 cells (Fig. 6G, H). To assess the effect of lactate on nucleus translocation of NF-κB p65, confocal microscopy analysis showed that lactate inhibited NF-κB p65 nuclear translocation mediated by TNF-α (Fig. 6I).

NF-κB is a major proinflammatory modulator. In our study we observed that lactate promoted Galectin-9 expression while inhibited NF-κB p65 signaling. So we speculated that NF-κB might have the function of transcription inhibition. It has been reported that HDAC3, a component in the corepressor complex, was a co-inhibitor of NF-κB [21]. We speculated that HDAC3 might play a role in the transcription inhibition of NF-κB. Immunoprecipitation assay revealed that HDAC3 bound to NF-κB p65 after TNF-α stimulation in HN4 and Cal27 cells (Fig. 6J). Knockdown of HDAC3 reversed the inhibition of Galectin-9 mediated by TNF-α (Figs. S16 and 6K) and BetA (Fig. 6L) in HN4 cells. BetA suppressed the transcription activity of *LGALS9* encoding the Galectin-9 protein, which was reversed after HDAC3 silencing (Fig. 6M). We focused on possible transcriptional regulation of Galectin-9 by NF-κB p65 and investigated whether NF-κB p65 was able to bind the promoter of Galectin-9. Database mining indicated that the Galectin-9 promoter presented a putative binding region for NF-κB p65 (Fig. 6N). Compared with TNF-α group, knockdown of HDAC3 can effectively promote the enrichment of NF-κB p65 in promoter regions of Galectin-9 (Figs. 6O and S17). The existence of HDAC3 leads to the suppression of NF-κB p65 transcriptional activity. In addition, we used RFPG966, the specific inhibitor of HDAC3, to detect its effect on tumorigenesis to find that RFPG966 significantly promoted cell colony formation and increased the number of invasion and migration cells in vitro and in vivo (Fig. S18). These results demonstrated that HDAC3 bound and restrict the transcription of NF-κB p65 to Galectin-9, which explained that lactate-induced Galectin-9 expression by NF-κB p65 inhibition.

### Overexpression of PKM2 correlates with lactate dehydrogenase and Galectin-9 expression in HNSCC tissues

**Fig. 7:**
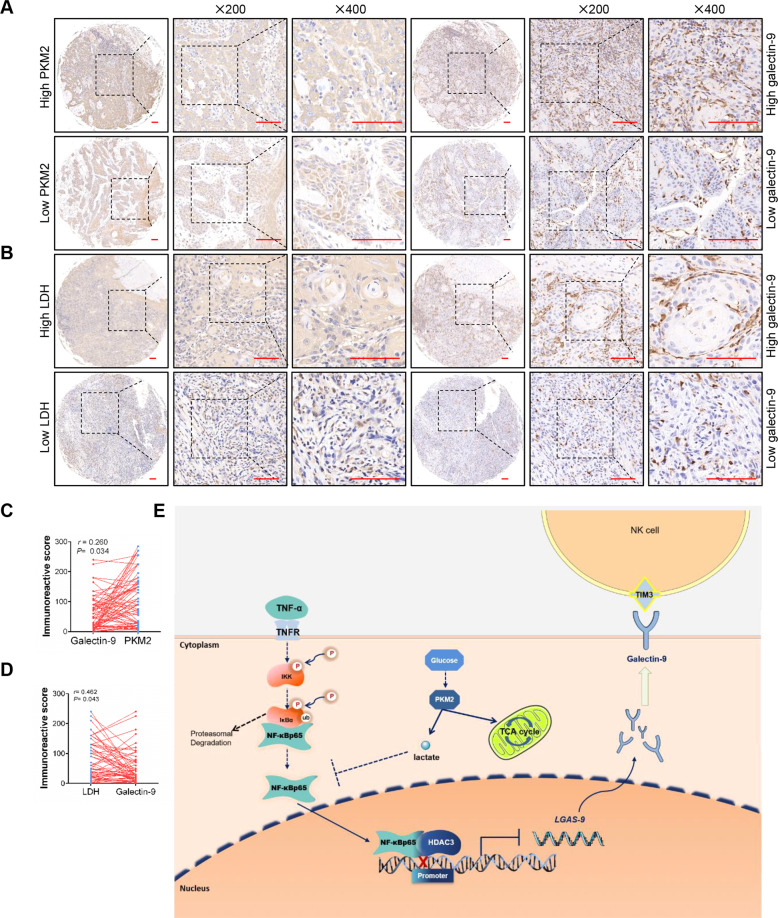
Overexpression of PKM2 correlates with Galectin-9 expression in HNSCC tissues. **A**, **B** Representative immunohistochemistry images of PKM2, Galectin-9, and LDH in HNSCC tissues were shown. **C**, **D** Correlation between PKM2, LDH, and Galectin-9 expression was analyzed in HNSCC patients. **E** Schematic diagram showed that lactate secreted by PKM2 upregulation promoted Galectin-9-mediated immunosuppression through inhibiting NF-κB pathway in HNSCC. Bar: 50 μm *P < 0.05, **P < 0.01.

To analyze whether lactate induced by PKM2 upregulation had an effect on immunosuppression, both PKM2 and Galectin‑9 were detected in paired HNSCC tissues. A positive correlation between PKM2 and Galectin-9 expression was observed in HNSCC tissues (Fig. 7A, C). Moreover, there was also a positive correlation between PKM2 and other immunosuppression-related molecules, CD274 (encoding PD-L1), HLA-E and FGL1 in HNSCC datasets (Fig. S19). Moreover, the IRS of Galectin-9 and LDH in HNSCC patients was higher than that in normal controls (Fig. S20). The expression level of Galectin‑9 correlated with pathological grade (Fig. S15, *P* = 0.0012), but there was no correlation between Galectin‑9 expression and other factors, including advanced TNM stage, gender, and age (Fig. S19). LDH is essential for the conversion of pyruvate into lactate. A positive correlation between LDH and PKM2 and Galectin‑9 was observed in the HNSCC tissues (Figs. 7B, D and S21). To sum up, this study demonstrates that PKM2-induced lactate production promotes Galectin-9 expression by inhibition of transcriptional suppression complex forming by NF-κB P65 and HDAC3 in HNSCC (Fig. 7E).

### Knockdown of PKM2 expression inhibits tumor proliferation and lung metastasis in vivo

**Fig. 8:**
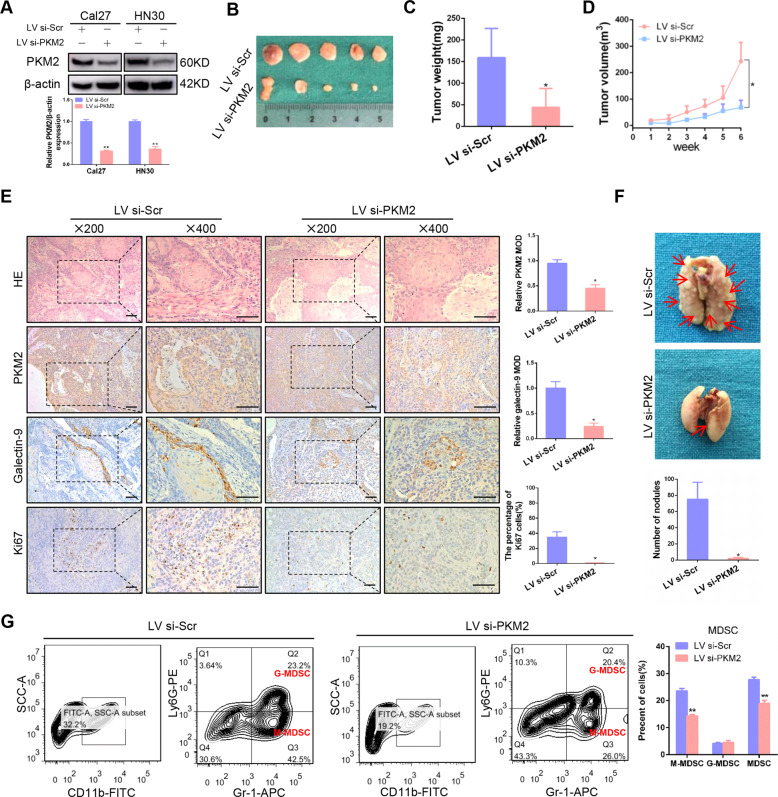
Knockdown PKM2 expression inhibits tumor proliferation and lung metastasis in vivo. **A** PKM2 expression was detected in Cal27 and HN30 after lentivirus stable transfection. **B** Images of tumors derived from the xenograft model were shown. **C** Tumor weight was measured after excision from mice. **D** The tumor volume was measured and analyzed once a week. **E** Representative images of the HE, PKM2, Galectin-9, and Ki-67 staining by IHC in tumor sections were shown. **F** Representative images of lung metastasis in each group were shown and the number of metastasis nodules was compared in two groups. **G** The infiltration of MDSC in mice tumor microenvironment was shown and analyzed. Bar: 50 μm. *P < 0.05, **P < 0.01.

To further confirm the above in vitro results, PKM2 knockdown model was established in vivo. The lentivirus transfection efficiency was confirmed in Cal27 and HN30 cells (Fig. 8A). Knockdown of PKM2 expression notably inhibited tumor growth in the xenograft model (Figs. 8B–D and S22). Moreover, lower expression of PKM2, Galectin-9, and ki-67 was observed in xenograft tissue from the PKM2 knockdown group compared to the control (Fig. 8E). In addition, the percentage of apoptotic cells increased in PKM2 expression in the PKM2 knockdown group (Fig. S23). The lung metastasis assay showed that knockdown of PKM2 expression significantly inhibited lung metastasis, which was diagnosed using HE staining. The number of metastasis nodules was notably decreased after PKM2 knockdown (Figs. 8F and S24). In addition, a negative correlation between Galectin-9 and E-cadherin expression was observed in HNSCC tissues (Fig. S25), which suggested the closed relationship between immune escape and distant metastasis. One of the mechanisms by which Galectin-9 promotes immunosuppression is via inducing proliferation of myeloid derived suppressor cells (MDSC). We observed that PKM2 silencing reduced the infiltration of MDSC in xenograft tumor microenvironment (Fig. 8G). These results demonstrated that PKM2 played an oncogenic role during tumourgenesis and metastasis via immunosuppression in HNSCC.

## Discussion

Nowadays metabolism reprogramming and immune escape have become a research focus. We demonstrated that PKM2 acted not only as an enzyme for glycolysis but as an oncogene driving tumor progression in HNSCC. These results showed that PKM2 promoted tumor progression and Galectin-9-mediated immunosuppression by lactate production in HNSCC. Our study makes a novel connection between tumor metabolites and immune checkpoints in HNSCC.

PKM2 has been reported to act as an oncogene in several carcinomas, but its oncogenic mechanism is still obscure. The *PKM* gene can be alternatively spliced to either the *PKM1* or *PKM2* transcript, while the PKM2 isoform is almost invariably expressed in cancer cells [22]. PKM2 attracted our attention because it is the last rate-limiting enzyme in glycolysis. However, accumulating evidence suggested that PKM2 was not just an enzyme but had diverse functions during tumor progression [23]. PKM2 overexpression has been reported in several types of human cancers. In cardiovascular disease, PKM2 serves as a molecular integrator of metabolic dysfunction, oxidative stress and tissue inflammation [24]. In pancreatic adenocarcinoma, abrogation of PKM2 resulted in impaired proliferation and increased apoptosis [25]. Indeed, PKM2 overexpression in HNSCC patients has been reported, but there was no further study concerning the biological function of PKM2 and its carcinogenesis mechanism [26–28]. In our study, we also observed that overexpression of PKM2 indicated poor prognosis in HNSCC patients. In our study, we found that knockdown of PKM2 promoted G1 arrest and induced apoptosis, along with ERK1/2 activation. ERK1/2 cascades are responsible for basic cellular processes, including cell proliferation and differentiation [29]. However, it has been reported that sustained activation of ERK1/2 leads to G1 phase arrest [30]. We thus speculated that the phosphorylation of ERK1/2 after 48 h stimulation was the reason why the cells are arrested at G1 phase. Moreover, we thoroughly investigated its effect on proliferation and metastasis capacity using silencing and an ectopic expression model in vivo and in vitro. These results deepen our understanding of oncogenic characteristics mediated by PKM2 overexpression in HNSCC.

In this study, we found that overexpression of PKM2 could acidify the tumor microenvironment by lactate secretion. Lactate, as a malignant biomarker, is strongly produced within the tumor microenvironment [31]. It plays an important role in cancer aggressiveness and poor survival [32, 33]. Lactate not only affects epigenetic modification but also harbors hormone-like properties [34]. It was also observed that lactate had a paracrine effect on hypoxia-inducible factor 1 and vascular endothelial growth factor signaling [35]. Moreover, lactate import could augment the aggressive features of cancer cells [36]. In our study, lactate notably promoted cell migration and invasion capacity in HNSCC.

It has been reported that lactate-mediated motility inhibition inactivated CD4 + and CD8 + T cell subsets [37]. Furthermore, tumor-derived lactate inhibits the function of NK cells [38]. In addition, acidification of the microenvironment has been shown to disturb inflammatory cytokine production [39]. These studies indicated that lactate plays a critical role in immune escape. A recent study showed that lactate activated the programmed death ligand-1 PD-L1/PD-1 immune checkpoint and impaired T cell function [40]. Meanwhile, PKM2 was required for the expression of PD-L1 [41]. This indicated that lactate might regulate the expression of immunosuppression molecules. Galectin-9, a ligand for TIM-3, impairs innate immunity by downregulating NK cell function [42]. Our results demonstrated that PKM2 could enhance Galectin-9 expression by promoting the secretion of lactate. We also found a positive correlation between PKM2 and other immune checkpoint molecules, such as CD274, FGL1, and HLA-E, in the TCGA database. Unfortunately, PKM2 failed to upregulate PD-L1 protein expression in HN4 and Cal27 cells (data not shown). Moreover, the MCT1 inhibitor could regulate PKM2-induced Galectin-9 expression through disturbing lactate transport, which suggested that lactate secretion played a crucial role in PKM2-induced immunosuppression in HNSCC. Moreover, lactate secretion promotes immunosuppression formation via NF-κB signaling in HNSCC. Lactate can suppress the T and NK cells leading to tumor immune escape. Inflammation process is associated with activation of the NF-κB. But our results showed that lactate inhibited NF-κB p65 and thereby increased the expression of Galectin-9. It needed to further elucidate how NF-κB p65 inhibits Galectin-9 expression. NF-κB molecules have been shown to interact with histone deacetylase to exert the function of transcription inhibition [43]. Our data indicated that knockdown HDAC3 can reverse the inhibition of NF-κB on Galectin-9 promoter, which releases the transcription activation capacity of NF-κB p65.

## Conclusion

In summary, this study demonstrated that oncogenic PKM2 drive tumor progression and Galectin-9-mediated immunosuppression by lactate production. Moreover, lactate inhibited NF-κB signaling and it acted as a transcription suppressor through binding to HDAC3 in HNSCC. The PKM2/lactate/NF-κB/Galectin-9 axis represented a novel bridge and potential therapeutic target between a metabolite and immunosuppression in HNSCC.

## Supplementary information

Revised supplementary figure

Revised supplementary Legends
