# Supplementary material for: Lactate secreted by PKM2 upregulation promotes Galectin-9-mediated immunosuppression via inhibiting NF-κB pathway in HNSCC
Source: Cell Death Dis. 2021 Jul 21;12(8):725. doi: 10.1038/s41419-021-03990-4 (PMC8295286; doi:10.1038/s41419-021-03990-4)
Supplement: Supplementary file 2 — Revised supplementary Legends [file 41419_2021_3990_MOESM2_ESM.docx]

**Supplementary Figure Legends**

Supplementary Fig. 1. Representative images of negative and positive expression of nuclear PKM2 in HNSCC were shown. Bar: 50 μm.

Supplementary Fig. 2. Quantitative statistical analysis of western blot of Figure 1I.

Supplementary Fig. 3. Quantitative statistical analysis of western blot of Figure 1K.

Supplementary Fig. 4. ERK1/2, p-ERK1/2, AKT and p-AKT were detected after PKM2-specific siRNA transfection for 48 h. ERK1/2 and p-ERK1/2 levels were detected after PKM2 overexpression for 48 h.

Supplementary Fig. 5. Migration and invasion capability were analyzed using transwell assay after siRNA transfection for 24 h in HN30 cells.

Supplementary Fig. 6. An electronic pH detector was used to measure pH in the medium supernatant of HN4 and HN30 after plasmid transfection for 48 h.

Supplementary Fig. 7. The morphology of HN4 and HN30 was detected by Actin-Tracker Green after 50 mM lactate stimulation for 24 h. Bar:200 μm.

Supplementary Fig. 8. Live cell analysis of HN4 and HN30 was detected after treated with 50 mM lactate for 24 h. Bar:200 μm.

Supplementary Fig. 9. E-cadherin levels were detected after the indicated lactate stimulation for 24 h.

Supplementary Fig. 10. Quantitative statistical analysis of western blot of Figure 5F.

Supplementary Fig. 11. Galectin-9 concentration in medium supernatant was detected after transfected with PKM2-expression plasmid using ELISA.

Supplementary Fig. 12. The knockdown efficiency of Galectin-9 was validated after transfection for 48 h.

Supplementary Fig. 13. Transfected cells were seeded at a density of 1 × 10^3^ cells per well in a 96-well plate and then incubated with NK cells for 4 h at various (E:T) ratios as indicated. The specific lysis was analyzed by the LDH kit.

Supplementary Fig. 14. IL-1β and IL-6 concentration were detected after transfected with PKM2-expression plasmid using ELISA.

Supplementary Fig. 15. p-AMPK, p-LATS and p-YAP expression were detected after lactate stimulation in HNSCC cells for 24 h.

Supplementary Fig. 16. Transfection efficiency was detected using Western blot after HDAC3-specific siRNA transfection for 48 h.

Supplementary Fig. 17. ChIP assays were performed using isotype IgG antibody after treatment with 50μg/ml TNF-α and HDAC3-specific siRNA.

Supplementary Fig. 18. HDAC3 inhibitor, RFPG966, promoted tumorigenesis and metastasis. A, B. Migration and invasion ability was analyzed using transwell assay after treatment with the RFPG966, the specific inhibitor of HDAC3. C. Colony formation was performed to detect proliferation ability after treatment with the RFPG966. D, E. Representative images of mice and tumors derived from the xenograft model were shown. F. Tumor weight was measured after excision from mice. G. Images of HE staining by IHC in tumor sections were shown. bar: 100 μm.

Supplementary Fig. 19. The correlation between PKM2, CD274, FGL1 and HLA-E expression in HNSCC was analyzed from TCGA dataset.

Supplementary Fig. 20. IRS of Galectin-9 and LDH in HNSCC patients (n=70) and normal controls (n=18) was analyzed. The correlation between Galectin-9 expression and pathological grade, TNM stage, gender and age were analyzed in HNSCC patients.

Supplementary Fig. 21. Representative images and correlation of PKM2 and LDH in HNSCC tissues are shown. bar: 50 μm.

Supplementary Fig. 22. Xenograft tumors were shown after lentivirus-mediated PKM2 knockdown model.

Supplementary Fig. 23. TUNEL staining was performed to measure the apoptotic cells in tumor sections. bar: 50 μm.

Supplementary Fig. 24. Lung metastasis nodes were diagnosed by pathological examination. bar: 50 μm.

Supplementary Fig. 25. Representative images and correlation between Galectin-9 and E-cadherin in HNSCC tissues are shown. bar: 50 μm.
